# Fatigue and cancer: causes, prevalence and treatment approaches

**DOI:** 10.1038/sj.bjc.6602012

**Published:** 2004-07-06

**Authors:** L I Wagner, D Cella

**Affiliations:** 1 Center on Outcomes, Research and Education, Evanston Northwestern Healthcare, Robert H Lurie Comprehensive Cancer Center of Northwestern University, Northwestern University Feinberg Medical School, Chicago, IL, USA

**Keywords:** cancer-related fatigue, cancer symptom management

## Abstract

Fatigue is the most prevalent cancer-related symptom and has a significant adverse impact on patients' functional ability and quality of life. Hypotheses regarding the aetiology of cancer-related fatigue are discussed, and clinical practice guidelines for the evaluation and management of oncology patients with fatigue are reviewed. Both nonpharmacologic and pharmacologic strategies for the management of fatigue are summarised.

Publications on cancer-related fatigue were identified through Medline searches based on keywords (search terms cancer and fatigue, cancer-related fatigue, cancer and fatigue and intervention, cancer and fatigue and treatment) and based on authors who have published many articles on cancer-related fatigue or cancer-related symptom management, limited to 1966–2004. A narrative review of reference sections from recently published journal articles, review articles, and textbook chapters was also conducted. The brief nature of this review article does not allow for a comprehensive presentation of results from these searches. A hierarchical approach was used to select publications for inclusion in this review. When possible, priority was given to publications that were based on the most methodologically sound research studies. As cancer-related fatigue is still a relatively new area for research, exploratory or pilot results are cited when results from larger studies are not available.

Owing to increasingly earlier detection and improving therapies, the life expectancy of people with cancer has been increasing. As a result, cancer has become a chronic illness for most patients. Understanding the aetiology of cancer-related symptoms and the identification of efficacious clinical management strategies are therefore needed. Fatigue is the most prevalent cancer-related symptom and is associated with significant morbidity, functional impairments and reduced quality of life. Therefore, the effective management of fatigue would significantly reduce disease burden associated with cancer and its treatments.

Research in the area of cancer-related fatigue lags behind attention that has been given to cancer pain, nausea and vomiting (Curt and Johnston, 2003; Morrow *et al*, 2002). Recognising this issue, the US National Institutes of Health (NIH) recently held a State-of-the-Science Conference on Symptom Management in Cancer to discuss the clinical management of pain, depression and fatigue as priorities for advancing clinical care (National Institutes of Health, 2002).

## PREVALENCE OF CANCER-RELATED FATIGUE

Fatigue has been described as a nearly universal symptom among cancer patients by the National Comprehensive Cancer Network (NCCN) Cancer-Related Fatigue Panel (Mock *et al*, 2000). Depending on the patient sample and methodology employed, it is estimated that approximately 60–96% of cancer patients who are undergoing cancer treatments experience fatigue (Cella, 1998). Fatigue is extremely common among cancer patients undergoing cytotoxic chemotherapy, radiation therapy, bone marrow transplant or treatment with biological response modifiers (Irvine *et al*, 1994; Dean *et al*, 1995; Hann *et al*, 1999; Jacobsen *et al*, 1999). For a significant number of patients, fatigue persists after treatment is completed. Broeckel *et al* (1998) examined a sample of disease-free breast cancer patients who were a mean of 16 months postchemotherapy, and found patients' average fatigue to be 50% higher than that reported by a comparison group of women who did not have cancer. Bower *et al* (2003) assessed breast cancer survivors who were an average of 2.8–3 years postdiagnosis and found that survivors' average level of fatigue was comparable to that of age-matched women in the general population. Breast cancer survivors' fatigue scores were higher than a demographically and socioeconomically similar healthy group of women at risk for breast cancer.

## AETIOLOGY OF CANCER-RELATED FATIGUE

**Figure 1:**
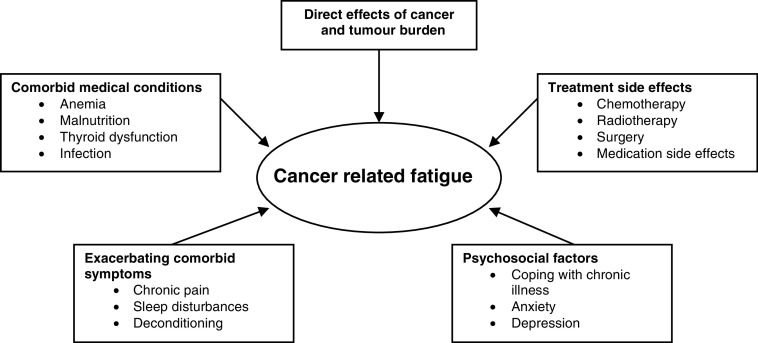
Causes of fatigue (Atkinson *et al*, 2002; Cella *et al*, 1998; Portenoy and Itri, 1999).

The pathophysiology of fatigue is poorly understood and few basic research studies have been conducted to investigate the physiological mechanisms underlying cancer-related fatigue (Gutstein, 2001). Identifying the aetiological factors that contribute to fatigue often proves to be complicated, as multiple causes typically coexist and may have additive effects. Figure 1

depicts the most commonly identified causes for cancer-related fatigue. Often cited factors include the direct effects of cancer, treatment side effects, comorbid medical conditions, exacerbating comorbid symptoms and psychosocial factors.

Morrow *et al* (2002) reviewed the evidence supporting four hypotheses for cancer-related fatigue: (1) anemia, (2) abnormalities in adenosine triphosphate, (3) vagal afferent activation and (4) the interaction of cytokines and serotonin. The authors reviewed research pertaining to each hypothesis and concluded by highlighting findings from various independent studies that have implicated cytokines, 5-HT, and the hypothalamic–pituitary axis in the development of cancer-related fatigue. Kurzrock (2001) has discussed the role of cytokine dysregulation in cancer-related fatigue and other conditions that are commonly associated with fatigue, such as anemia, cachexia, fever, infection and depression. Specifically, Kurzrock (2001) discussed erythropoietin, interleukin, tumour necrosis factor and interferon. Bower *et al* (2003) compared 20 breast cancer survivors with enduring fatigue to 19 nonfatigued breast cancer survivors and their findings suggest that persistent fatigue may be associated with a T-cell mediated inflammatory process. Several models have been proposed for the pathophysiology of fatigue and promising findings have been reported in support of these models. Research in this area is preliminary and it is clear that larger, well-designed studies are required to definitively pinpoint the causes of fatigue and the associated clinical implications.

## DIAGNOSTIC CRITERIA FOR CANCER-RELATED FATIGUE

The Fatigue Coalition, a multidisciplinary group of medical practitioners, researchers and patient advocates developed diagnostic criteria for cancer-related fatigue, with the goal of facilitating research and treatment planning through the availability of diagnostic criteria (Cella *et al*, 1998). The International Classification of Diseases-10 (ICD-10) criteria for cancer-related fatigue require ‘significant fatigue, diminished energy, or increased need to rest, disproportionate to any recent change in activity level’ to be present every day or nearly every day during the same 2-week period in the past month, as well as the presence of additional symptoms. A few examples of the additional symptoms that count toward a diagnosis of cancer-related fatigue include generalised weakness, diminished concentration, insomnia or hypersomnia and unrestorative sleep (for full criteria, see Cella *et al*, 1998). Since these are proposed criteria, there may be experts on cancer-related fatigue who would disagree with some of the additional symptoms as counting toward a diagnosis. Symptoms must cause clinically significant distress or impairment, be associated with cancer or cancer therapy, and cannot be primarily due to a comorbid psychiatric disorder. A few examples of exclusionary psychiatric conditions are provided in the criteria. One of the challenges in using these criteria to assess patients is that when a psychiatric diagnosis is present, the extent to which fatigue can be attributed to the psychiatric disorder is at the discretion of the clinician and can be difficult to determine given the overlap in symptomatology.

To evaluate these criteria, Cella *et al* (2001) interviewed 379 patients who received chemotherapy or chemotherapy plus radiation therapy and administered diagnostic criteria. A total of 37% of respondents reported at least 2 weeks of fatigue in the previous month and 17% of respondents met criteria for cancer-related fatigue. Sadler *et al* (2002) administered diagnostic criteria to 51 patients undergoing blood or bone marrow transplantation and found that 21% met criteria for cancer-related fatigue. Comparisons among independent raters demonstrated high rates of reliability. Patients meeting criteria reported higher levels of fatigue severity, frequency, pervasiveness, and interference with quality of life and poorer role functioning, less vitality and more depressive symptoms. These results support the reliability and validity of the proposed criteria for cancer-related fatigue.

These estimates are much lower than previously reported rates for the prevalence of fatigue. Many published reports on the prevalence of cancer-related fatigue include individuals who report having fatigue in prevalence estimates, regardless of the severity of fatigue. The use of diagnostic criteria represents a more stringent approach to estimating the prevalence of cancer-related fatigue, thus prevalence estimates based on these criteria are lower and the cases that are identified include participants with greater severity of fatigue and associated functional impairments. Given the overlap with major depressive disorder, diagnostic criteria for cancer-related fatigue may be useful in identifying patients with fatigue who may respond to antidepressant therapy (Jacobsen *et al*, 2003).

## CLINICAL SIGNIFICANCE OF CANCER-RELATED FATIGUE

Cancer-related fatigue significantly impairs patients' quality of life and ability to function. Based on a sample of 397 patients, 91% of respondents with fatigue reported that fatigue prevented them from leading a ‘normal’ life, 88% reported an alteration in their daily routine due to fatigue, and of patients who were employed 75% changed their employment status because of their fatigue (Curt *et al*, 2000). Vogelzang *et al* (1997) interviewed 419 cancer patients and patients reported that *fatigue* adversely affected their daily lives more than pain (61% *vs* 19%). However, in a comparable survey of oncologists, respondents believed that *pain* adversely affected their patients more than fatigue (61, 37%, respectively). In addition to interfering with patients' quality of life and functional status, cancer-related fatigue may also disrupt treatments for cancer. Vogelzang *et al* (1997) found that approximately one-third of patients interviewed reported that fatigue impacted patients' concerns regarding their mortality or compromised their hope of fighting the illness. The relationship between symptom management and cancer treatment adherence has been identified as a priority for investigation by the US National Institutes of Health (2002).

## CLINICAL MANAGEMENT OF CANCER-RELATED FATIGUE

For clinical interventions to be effective, patients with fatigue who would benefit from treatment must first be identified. The extent to which fatigue is underassessed and undertreated has been documented. Vogelzang *et al* (1997) interviewed 419 cancer patients and found that among fatigued patients, 50% did not discuss treatment options for fatigue with their oncologists and only 27% reported that their oncologist offered treatment recommendations for their fatigue. Curt *et al* (2000) similarly reported that 40% of patients with fatigue did not receive any treatment recommendations. Clinical practice guidelines for the management of fatigue have been established (Portenoy and Itri, 1999; Mock *et al*, 2000). Interdisciplinary evaluation and treatment is recommended to adequately assess and manage the complex interaction of medical, psychological and social factors that impact fatigue characteristics and associated decrements in quality of life.

### Screening for fatigue

Depending on available resources, busy clinical settings may benefit from implementing screening tools to identify patients with more severe fatigue so that these patients can be targeted for in-depth assessment and treatment. The effectiveness of single item or brief measures to identify cancer patients who may benefit from assessment and treatment for fatigue has been demonstrated (Kirsh *et al*, 2001). National Comprehensive Cancer Network recommends the use of a single item to assess fatigue severity on a 0–10 scale. To identify the cutoff score on a 0–10 scale that maximises sensitivity and specificity in detecting cases of more severe fatigue, Wagner *et al* (2003a) administered a single-item screening for fatigue and a longer measure of fatigue (Functional Assessment of Cancer Therapy – Fatigue subscale, FACT-F) to 105 outpatients who were receiving chemotherapy. A cutoff of 5 was the optimal level for detecting ‘cases’ of clinically significant fatigue as defined by exceeding an established threshold for caseness on the FACT-F. The suggested cutoff of 5 can be used as a guide for clinical practice. Additional factors should be considered when determining the optimal cutoff score for a particular clinical setting. Resources that are available to further assess and treat patients with fatigue should be taken into account when deciding on a cutoff score to target patients for more in-depth assessment and treatment when indicated. Clinics with staff available to conduct follow-up assessment on identified patients could afford to set a low cutoff score to identify all patients who have fatigue regardless of severity, whereas clinical settings with limited resources may need to set a higher cutoff score to identify only those patients with moderately severe to severe fatigue for focused intervention.

### Assessing patients with clinically significant fatigue

Given the diverse aetiological factors that contribute to fatigue and its multidimensional nature, the comprehensive assessment of patients with fatigue is required for the development of effective treatment interventions. Clinical practice guidelines for fatigue assessment and management emphasise the need to evaluate (1) fatigue characteristics and (2) disease status and treatment. Guidelines also recommend obtaining a history and physical checkup and conducting laboratory studies as indicated to rule out common, treatable causes of fatigue, such as anemia or thyroid dysfunction (Portenoy and Itri, 1999; Mock *et al*, 2000). An in-depth fatigue assessment should include fatigue severity, temporal characteristics (e.g. onset, duration), exacerbating and alleviating factors, impact on functioning and quality of life, symptom-related distress, and other symptoms that commonly co-occur with fatigue such as pain, menopausal symptoms, sleep disturbances, depression and cognitive dysfunction. Since fatigue is a subjective sensation, it is important to use validated, standardised assessment instruments. Many standardised instruments have been developed for the assessment of fatigue and its impact on quality of life (for a review, see Wagner and Cella, 2001).

### Interventions for cancer-related fatigue

The brief nature of this article does not allow a comprehensive review of empirical support for interventions to treat cancer-related fatigue. Priority was given to randomised clinical trials (RCTs) with fatigue as a primary end point. For interventions with several published RCTs (e.g. psychosocial interventions), results from less methodologically rigorous studies were not presented. For interventions that have not been as extensively investigated, open-label studies and pilot studies are cited. Clinical practice guidelines developed by the NCCN Cancer-Related Fatigue Panel (Mock *et al*, 2000) through evidence-based and consensus review are used to guide the discussion.

National Comprehensive Cancer Network Cancer-Related Fatigue Guidelines recommend a two-stage approach to the treatment of cancer-related fatigue. The first step in treating fatigued cancer patients is to identify and address any treatable contributing factors that may be causing the fatigue. National Comprehensive Cancer Network guidelines identify several commonly observed treatable contributing factors, including pain, emotional distress, anemia, sleep disturbance, nutritional inadequacies, deconditioning due to decreased activity and comorbidities (e.g. infection, cardiac dysfunction, renal dysfunction). Significant improvements in quality of life have been demonstrated among anemic cancer patients after treatment with erythropoietic agents (Demetri *et al*, 1998; Cella *et al*, in press).

Based on NCCN guidelines, the second step involves the management of any residual fatigue that continues after treatable contributing factors have been resolved or fatigue that continues despite the lack of any identified treatable contributing factors. To place treatment strategies in the context of the patient's clinical status, separate algorithms are provided for patients receiving active cancer treatment, patients receiving disease-free long-term follow-up, and patients receiving care at the end of life. Fatigue management includes providing education and counselling to patients and their families, self-management strategies that patients can utilise, nonpharmacologic interventions and pharmacologic treatments. Education and counselling should emphasise that fatigue is commonly experienced by patients undergoing treatment for cancer and is not necessarily an indicator of disease progression (if appropriate). National Comprehensive Cancer Network guidelines identify several strategies that patients may find useful for managing their fatigue, including energy conservation techniques such as prioritising activities, pacing (alternating physically demanding with more sedentary activities), scheduling activities at times of peak energy, taking naps as long as they do not interfere with night-time sleep and following a structured daily routine. Distraction through engaging in pleasurable activities is also recommended. Barsevick *et al* (2004) randomised cancer patients who were initiating chemotherapy or radiotherapy to an energy conservation and activity management (ECAM) intervention (*n*=200) or to a control intervention that was similar in terms of time and attention (*n*=196). Both interventions included three telephone sessions with an oncology nurse during the first 3–5 weeks of treatment. A statistically significant difference in fatigue over time was reported, with ECAM participants reporting a greater decrease in fatigue over time compared to control participants. No differences in functional performance were observed.

National Comprehensive Cancer Network guidelines recommend the following nonpharmacologic interventions: exercise, restorative therapy, nutrition consultation, sleep hygiene and psychosocial interventions. Of these interventions, the effectiveness of exercise in reducing fatigue and increasing functional abilities has received the most empirical support. Table 1

**Table 1:** Nonpharmacological interventions: exercise

**Author**	**Sample characteristics**	**Sample size**	**Design**	**Type of exercise**	**Results**
Dimeo *et al* (1999)	Mixed (Hem and solid tumour)	59	RCT	Bed cycle ergometer (biking)	↓ Fatigue
Mock *et al* (2001)	Breast cancer Stage I–III	52	RCT	Home-based walking programme 4–5 × week^−1^ for 30 min	↓ Fatigue, ↑ walking ability
Mock *et al* (2002)	Breast cancer Stage I–III	111	RCT	Home-based walking programme 4–5 × week^−1^ for 30 min	↓ Fatigue, ↑ walking
Schwartz *et al* (2001)	Breast cancer Stage II	61	Within subjects	Home-based walking programme or patient choice 3–4 × week^−1^ for 15–30 min	↓ Fatigue, ↑ walking ability
Schwartz *et al* (2002)	Melanoma patients treated with interferon-a	12 + 16 historical controls	Quasi-experimental	Patient-selected 4 × week^−1^ for 15 min + methylphenidate 20 mg QD	↓ Fatigue, ↑ functional ability
Segal *et al* (2003)	Prostate cancer receiving androgen ablation	155	RCT	Resistance exercise	↓ Fatigue, ↑ quality of life, ↑ muscular fitness

RCT=randomised clinical trial.

summarises results from six studies that examined exercise to treat cancer-related fatigue, four of which were randomised, controlled trials. Reductions in fatigue were reported from all six trials. Types of exercise included a home-based walking programme, a bed cycle ergometer, resistance exercise or patient-selected exercise. It is important to highlight the strength of the empirical support for exercise in treating cancer-related fatigue, since the most common treatment recommendation given to patients by their health care providers was bed rest and relaxation (Curt *et al*, 2000). According to NCCN guidelines, contraindications to exercise include bone metastases, neutropenia, thrombocytopenia and fever.

Psychosocial interventions have also received empirical support for the treatment of cancer-related fatigue, as demonstrated in Table 2

**Table 2:** Nonpharmalogical interventions: psychosocial

**Author**	**Sample characteristics**	**Sample size**	**Design**	**Type of intervention**	**Results**
Spiegel *et al* (1981)	Breast cancer Stage IV	86	RCT	Support group weekly 1 year	↓ Fatigue, anxiety, mood disturbance
Forester *et al* (1985)	Mixed cancer in radiation therapy	100	RCT	Individual psychotherapy 10 weeks	↓ Fatigue, emotional symptoms, physical symptoms
Fawzy *et al* (1990)	Melanoma postsurgery Stage I–II	66	RCT	Support group including education and stress management 6 weeks	↓ Fatigue, depression, mood disturbance
Fawzy (1995)	Melanoma Stage I–II	61	RCT	Individual education and RN support 3 h	↓ Fatigue, anxiety, mood disturbance
Gaston-Johansson *et al* (2000)	Autologous bone marrow transplantation	110	RCT	Coping strategy programme	↓ Fatigue and nausea
Given *et al* (2004)	Mixed solid tumour and lymphoma	237	RCT	Tailored behavioural intervention 8 weeks	↓ Fatigue and pain
Jacobsen *et al* (2002)	Mixed cancer in chemotherapy	411	RCT	Professionally or self-administered stress management training	↑ Vitality and mental health

RCT=randomised clinical trial.

. Seven randomised clinical trials to evaluate supportive interventions (group and individual), education and stress management groups, coping strategies training and behavioural interventions to assist patients with managing their fatigue have been published with impressive results. Given *et al* (2004) conducted a randomised clinical trial to evaluate a cognitive behavioural intervention to reduce cancer-related symptom burden. The investigators found that a 20-week (10 contacts) cognitive behavioural intervention demonstrated significant impact on severity of cancer-related symptoms after four contacts (10 weeks), and this reduction was maintained at 20 weeks among those patients who entered the trial with moderate to severe symptoms.

Pharmacological interventions may also be useful for the management of cancer-related fatigue, although research in this area is limited since the few studies that have been published have methodological shortcomings (Portenoy and Itri, 1999). Classes of potentially useful medications include erythropoietic agents (when anemia is present), psychostimulants, selective serotonin-reuptake inhibitors and low-dose corticosteroids. Dosing guidelines for erythropoietic agents to treat anemia have been established (see NCCN Cancer and Treatment-Related Anemia guidelines as an example, www.nccn.org).

Table 3

**Table 3:** Pharmalogic interventions: psychostimulants

**Author**	**Sample characteristics**	**Sample size**	**Design**	**Medication**	**Results**
Bruera *et al* (1987)	Advanced cancer with chronic pain	32	Open-label	Methylphenidate	↓ Psychomotor slowing, ↑ physical activity
Bruera *et al* (2003)	Advanced cancer	31	Open-label	Methylphenidate	↓ Fatigue, ↑ quality of life, ↑ sleep quality
Wilwerding *et al* (1995)	Cancer patients receiving opioids	43	RCT	Methylphenidate	↓ Somnolence
Meyers *et al* (1998)	Primary brain tumour	30	Within subjects	Methylphenidate	↓ Psychomotor slowing, ↑ cognitive functioning
Rammohan *et al* (2002)	Multiple sclerosis	72	RCT	Modafinil	↓ Fatigue
Krupp *et al* (1995)	Multiple sclerosis	93	RCT	Amantadine, pemoline, or placebo	Amantadine:, ↓ fatigue, pemoline: fatigue severity same as placebo
Weinshenker *et al* (1992)	Multiple sclerosis	46	RCT	Pemoline	↓ Fatigue (*P*=0.06)

RCT=randomised clinical trial.

summarises research on the efficacy of psychostimulants in treating fatigue among cancer patients, the elderly and patients with multiple sclerosis. Examples of psychostimulants include methylphenidate, modafinil, pemoline and dextroamphetamine. Controlled, randomised trials have yielded evidence to support the relative efficacy of psychostimulants in treating fatigue associated with opioid administration among cancer patients (Wilwerding *et al*, 1995). Bruera *et al* (2003) conducted a prospective open-label trial with advanced cancer outpatients who had fatigue > 4 (0–10 scale) to evaluate the effects of patient-controlled methylphenidate for cancer-related fatigue. FACIT-F (Functional Assessment of Chronic Illness Therapy – Fatigue) scores and Edmonton Symptom Assessment Scale (ESAS) scores showed significant improvement in fatigue after 7 days of treatment, with no reported serious side effects. Schwartz *et al* (2002) found that methylphenidate combined with exercise reduced fatigue among interferon-treated melanoma patients (see Table 1). The efficacy of modafinil in treating fatigue secondary to multiple sclerosis was demonstrated in a Phase II trial (Rammohan *et al*, 2002). A trial of modafinil to treat cancer-related fatigue is currently underway. Weinshenker *et al* (1992) evaluated pemoline for the treatment of fatigue among patients with multiple sclerosis and found a trend suggesting reduced fatigue among patients who received pemoline compared to placebo (*P*< 0.06). Krupp *et al* (1995) conducted a randomised, clinical trial to compare amantadine, pemoline, or placebo for the treatment of fatigue in patients with multiple sclerosis. Patients who received amantadine had significantly less fatigue compared to those receiving pemoline or placebo. However, patients receiving pemoline did not differ from patients who received placebo in reported fatigue severity.

National Comprehensive Cancer Network guidelines recommend antidepressants for fatigue management, particularly when depression is present. While the close association between fatigue and depression suggests that antidepressants may have a role in treating cancer-related fatigue, research in this area has not been supportive of this class of medications. Morrow *et al* (2003) conducted a randomised, double-blind trial comparing 244 patients who received paroxetine to 235 patients treated with placebo. Patients treated with paroxetine reported less depressive symptoms at follow-up; however, no differences in fatigue severity were observed.

Clinical observation and limited data from controlled trials suggests that low-dose cortisteroids may have a role in treating cancer-related fatigue, specifically dexamethasone and prednisone. Bruera *et al* (1990) demonstrated the efficacy of megestrol acetate to manage cachexia and multiple symptoms including fatigue among advanced cancer patients.

In sum, pharmacological approaches to the management of cancer-related hold promise, particularly psychostimulants. It is clear that randomised clinical trials with all of these agents are needed to advance the development of efficacious treatment approaches. Table 4

**Table 4:** Interventions: current status of empirical support for clinical management strategies for cancer-related fatigue

	**Empirical support**	**Demonstrated in other diseases**	**Additional research needed**
Exercise	X		
Psychosocial interventions	X		
Erythropoetic agents (if anemia is present)	X		
Psychostimulants		X	X
Selective serotonin reuptake inhibitors (SSRIs)			X
Low-dose corticosteroids		X	X

summarises the current status of empirical support for nonpharmacological and pharmacological fatigue management strategies.

## CONCLUSION

Fatigue is a symptom commonly associated with cancer and its treatments. The mechanisms underlying the development and maintenance of fatigue are poorly understood. Fatigue is multifactorial in its aetiology and manifestation. Empirical support for exercise and psychosocial interventions for fatigue have been reported. Pharmacological treatments currently in use are based on limited research studies. Short-term goals of clinical research on cancer-related fatigue should focus on an improved understanding of the aetiology of fatigue, relationships between fatigue and co-occurring symptoms, and evaluating treatment interventions (National Institutes of Health, 2002; Wagner *et al*, 2003b). Well-designed clinical trials are urgently needed for the development of empirically supported fatigue management strategies to reduce the symptom burden associated with cancer.
